# Association of Youth Age at Exposure to Household Dysfunction With Outcomes in Early Adulthood

**DOI:** 10.1001/jamanetworkopen.2020.32769

**Published:** 2021-01-07

**Authors:** Signe Hald Andersen

**Affiliations:** 1 Rockwool Foundation Research Unit, Copenhagen, Denmark

## Abstract

**Importance:**

The current focus on the association of negative experiences in early childhood with adverse outcomes later in life is based on limited empirical evidence.

**Objective:**

To evaluate whether age at exposure to negative experiences in childhood and adolescence is associated with outcomes in early adulthood.

**Design, Setting, and Participants:**

This cohort study used population data from administrative sources for all Danish individuals born between 1987 and 1995 who were living in Denmark at 19 years of age. Data were analyzed in July 2020.

**Exposures:**

Exposure to 6 household dysfunction items (HDIs) from birth to 17 years of age by age group. Age groups were as follows: 0 to 2 years (early childhood), 3 to 5 years (preschool), 6 to 12 years (mid-childhood), and 13 to 17 years (early adolescence). The 6 items were parents’ unemployment, incarceration, mental disorders, death, and divorce and the child’s foster care experiences.

**Main Outcomes and Measures:**

Mental disorders, low educational attainment, disconnection from education and the labor market, and criminal charges. A fixed-effects model was used to estimate the dose-response and age-specific associations between HDI exposure and the collated outcome measure.

**Results:**

The study sample included 605 344 individuals observed from birth to 19 years of age (mean [SD] birth year, 1991 [2.56] years; range, 1987-1995; 335 725 [55%] male). Overall, 278 115 individuals (45.94%) were exposed to 1 or more of the 6 HDIs from birth to 17 years of age. Exposure was most prevalent at 1 year of age (exposure rate, 11.3%), and parental unemployment was the most common HDI (15.5% observed in mid-childhood). The risk of experiencing the 4 outcomes was monotonically associated with the number of HDIs. For example, experiencing 1 HDI between birth and 17 years of age was associated with an increased risk of experiencing an adverse outcome by approximately 1.0 percentage point (β = 0.011; 95% CI, 0.010-0.012; *P* < .001). Similarly, the risk of experiencing adverse outcomes varied significantly in association with age at exposure. Exposure during early adolescence was more strongly associated with adverse outcomes than was exposure during early childhood (increased risk of 5.8 percentage points [β = 0.058; 95% CI, 0.052-0.063; *P* < .001] vs 1.0 [β = 0.010; 95% CI, 0.004-0.015; *P* = .001]).

**Conclusions and Relevance:**

In this cohort study, exposure to negative experiences in early adolescence was more strongly associated with later adverse outcomes than was exposure at other points in childhood. Knowledge of age-specific associations is important information for policy makers who need to prioritize resources targeting disadvantaged children and youths.

## Key Points

**Question:**

Is age at exposure to negative experiences in childhood and adolescence associated with subsequent adverse outcomes?

**Findings:**

In this cohort study of data on 605 344 individuals aged 19 years from Denmark, exposure to negative experiences in early adolescence was more strongly associated with later adverse outcomes than was exposure in early childhood.

**Meaning:**

The findings suggest that policy interventions targeting individuals exposed to negative experiences during childhood should focus on individuals exposed to negative experiences in adolescence.

## Teaser

This cohort study examines the association between age at exposure to household dysfunction during childhood and adolescence and outcomes in early adulthood.

## Introduction

Social scientists are currently advocating the importance of the association of positive and negative experiences in early childhood with biological, behavioral, and social outcomes in part because of heightened brain sensitivity from conception to age 3 years.^1,2,3^ In response, policy makers, child educators, and others have focused on the first years of childhood for securing cognitive functioning and physical and mental health in the adult population.^4,5^ However, insights from neuroscience provide a second perspective that adolescence is also a sensitive period in brain development, implying that experiences during this period are similarly crucial for later outcomes.^6^ This perspective has reached the United Nations Children's Fund (UNICEF), which now describes adolescence as an important second window of opportunity for developing appropriate interventions.^7^

Empirical evidence on age-specific effects of positive or negative experiences is not sufficient for use in building policies. Most of the existing evidence relies on small samples,^8^ data from a limited period in childhood, and retrospective measures of experiences in childhood that are imprecise because of recollection bias.^9,10^ Furthermore, most studies have shown an association of age at first exposure^8^ or the dose response in the number of experiences during childhood with later outcomes^9,10,11,12^ rather than age-specific effects of such experiences. This third and last perspective on the dose-response association reflects the hypothesis that chronic or recurrent stress is associated with negative outcomes in physiological and psychological functioning (allostasis^13^). In summary, the literature has shown 3 perspectives on when exposure to positive or negative experiences may be associated with outcomes later in life: (1) the early childhood critical period, (2) the adolescent critical period, and the (3) dose-response association. Each perspective draws on equally plausible explanations of brain development and the consequences of stress; however, the empirical evidence in terms of their relative importance is scarce.

The hypothesis of this study was that associations between exposure to negative experiences in childhood and later adverse outcomes vary by age at exposure. This study had 3 main objectives: (1) to assess the dose-response association between negative experiences during childhood and adolescence and outcomes later in life, (2) to examine the association between age at exposure to negative experiences and outcomes later in life, and (3) to examine the dose-response association by age at exposure.

## Methods

### Data

This cohort study used prospective population data for all Danish individuals born from 1987 to 1995 who were living in Denmark at 19 years of age (N = 605 344). All Danish residents have a unique personal number that identifies their interactions with the public sector (eg, when receiving welfare benefits, being incarcerated, or attending school) and several private institutions (eg, banks, private hospitals) and that enables family linkages. Statistics Denmark collects information on these interactions by the start and ending dates (since 1980) and grants access to this administrative data for research purposes. Data were analyzed in July 2020. The study was approved through a long-standing agreement between the Rockwool Foundation Research Unit and Statistics Denmark. Data were deidentified, and informed consent was waived following the Danish Data Protection Law, article 14(5b). This study followed the Strengthening the Reporting of Observational Studies in Epidemiology (STROBE) guideline.

### HDI Measures

With use of the data registers, I measured occurrences in childhood and adolescence of 6 household dysfunction items (HDIs) commonly recognized as factors associated with a range of later adverse outcomes^9,12,13,14,15,16,17,18,19^: (1) parental divorce, (2) prolonged unemployment (>9 months within a year) of 1 or both parents, (3) incarceration of the father, (4) inpatient treatment of a parent for mental illness, (5) foster care placement of the child, and (6) death of 1 or both parents (the eMethods in the Supplement gives definitions).

With this information, I constructed 3 different variables of HDI exposure. First, to assess the dose-response association between HDIs and later outcomes, I calculated the number of age-years of HDI exposure between birth and age 18 years for everyone in the sample. To account for dose, exposure to 2 different HDIs at a given age counted as exposure at 2 ages rather than just 1. Also, an HDI spanning multiple ages counted at each age that it affected.

Second, to assess the age-specific associations between HDI exposure and later outcomes, I constructed 4 binary variables that took the value 1 if the individual had been exposed to 1 or more HDIs in the following age groups: birth to 2 years, 3 to 5 years, 6 to 12 years, and 13 to 17 years. These age groups follow standard categories used in the literature and reflect knowledge of sensitive periods in brain formation.^8,20,21,22,23,24^

Third, to assess whether a dose-response association existed between HDI exposure and later outcomes within the 4 age groups, I constructed 4 count variables that measured duration of HDI exposure in years in the following age groups: birth to 2 years, 3 to 5 years, 6 to 12 years, and 13 to 17 years.

### Outcome Measures

In the analyses, I tested the association between HDI exposure and the risk of being diagnosed with a mental disorder, being charged with a criminal offense, being disconnected from education and the labor market, and not graduating from primary school. Each outcome measured the nature and success of the transition into adulthood, and these outcomes are among those needing to be reduced to sustain societies.^12,25^ All outcomes were measured at ages 18 and 19 years (eTable 3 in the Supplement gives definitions). The 4 outcome measures were correlated, although not strongly (eTable 3 in the Supplement), suggesting that they measure different dimensions. In the analyses, I used a collated outcome measure that took the value of 1 if the individual experienced 1 or more of these 4 outcomes.

**Figure 1.**
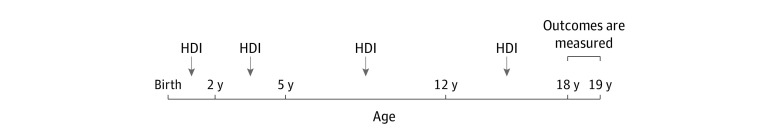
Data Structure for the Study Data are based on a birth cohort from 1987 to 1995. The vertical arrows indicate the age when household dysfunction item (HDI) exposure may occur.

Figure 1 shows the sample, data, and variables structure. Cohorts born from 1987 to 1995 were followed up, HDIs that occurred before age 18 years were measured, and outcomes at ages 18 and 19 years were observed.

### Statistical Analysis

I used a sibling fixed-effect model to estimate the dose-response and age-specific associations between HDI exposure and the collated outcome measure (eMethods in the Supplement). I also used the sibling fixed-effect model in the analysis of the dose-response association within age groups, and all models controlled for birth year, birth order, and sex (eTable 3 in the Supplement). From the models, I report the β coefficient, which is the change in the probability of experiencing the outcome variable for every 1-unit change in the HDI measure while holding all other variables in the model constant.

I conducted 3 sensitivity analyses. First, I evaluated whether the age-specific associations reflect proximity in time between exposure and outcome. I constructed 10 binary outcome variables that equaled 1 if the individual experienced the adverse outcome at each of the 10 years between age 20 and 29 years (eFigure 3 in the Supplement gives descriptive statistics). I included only the 1987 cohort (n = 59 283) because only this cohort was observable until age 29 years (eTable 1 and eFigures 1 and 2 in the Supplement give the HDI distribution). Second, the nature of problems that parents encounter varies across their lifetime, and because parents’ age confounds a child’s age, a child’s age-specific associations between HDIs and later adverse outcomes may mask parents’ age-specific variation in the problems. I addressed this concern by evaluating whether the associations between the HDI exposure and the adverse outcomes varied by mother’s age at childbirth in 4 subsamples defined by mother’s age at the time of the child’s birth (sample A: mother’s age <25 years [n = 123 096]; sample B: mother’s age, 25 to <30 years [n = 240 289]; sample C: mother’s age 30 to <35 years [n = 171 765]; and sample D: mother’s age ≥35 years [n = 70 194]). I used ordinary least squares for the first sensitivity analysis and the sibling fixed-effect model for the second and third sensitivity analyses. A 2-tailed *P* < .05 was considered significant. I used StataMP, version 15 (StataCorp LLC) for all analyses.

## Results

Of the 605 344 individuals in the study sample (mean [SD] birth year, 1991 [2.56] years; range, 1987-1995; 335 725 [55%] male), 278 115 (45.94%) had been exposed to 1 or more of the 6 HDIs between birth and 18 years of age (eTable 1 in the Supplement). The maximum number of HDIs experienced was 55 (eFigure 1 in the Supplement); a mean (SD) of 8.2% (27.4%) to 11.3% (31.7%) of individuals experienced 1 or more HDIs at each age (lowest at age 17 years and highest at age 1 year), and the cumulative proportion increased monotonically by age (eTable 1 in the Supplement). The 6 HDIs were correlated, although not strongly (eTable 2 in the Supplement). Parental unemployment and incarceration of the father were most strongly correlated (*r*, 0.1480-0.2152). Parental divorce and child foster care had the weakest correlation (*r*, −0.0043 to 0.0468).

The baseline mean (SD) risk for 4 adverse outcomes were as follows: being diagnosed with a mental disorder, 3.9% (17.9%); being charged with a criminal offense, 6.8% (25.2%); becoming disconnected from education and the labor market, 3.3% (17.9%); and not graduating from primary school, 2.0% (14.1%) (eTable 3 in the Supplement). The baseline risk of experiencing at least 1 outcome was 13.4% (the collated measure).

### Dose Response and Age at Exposure

**Figure 2.**
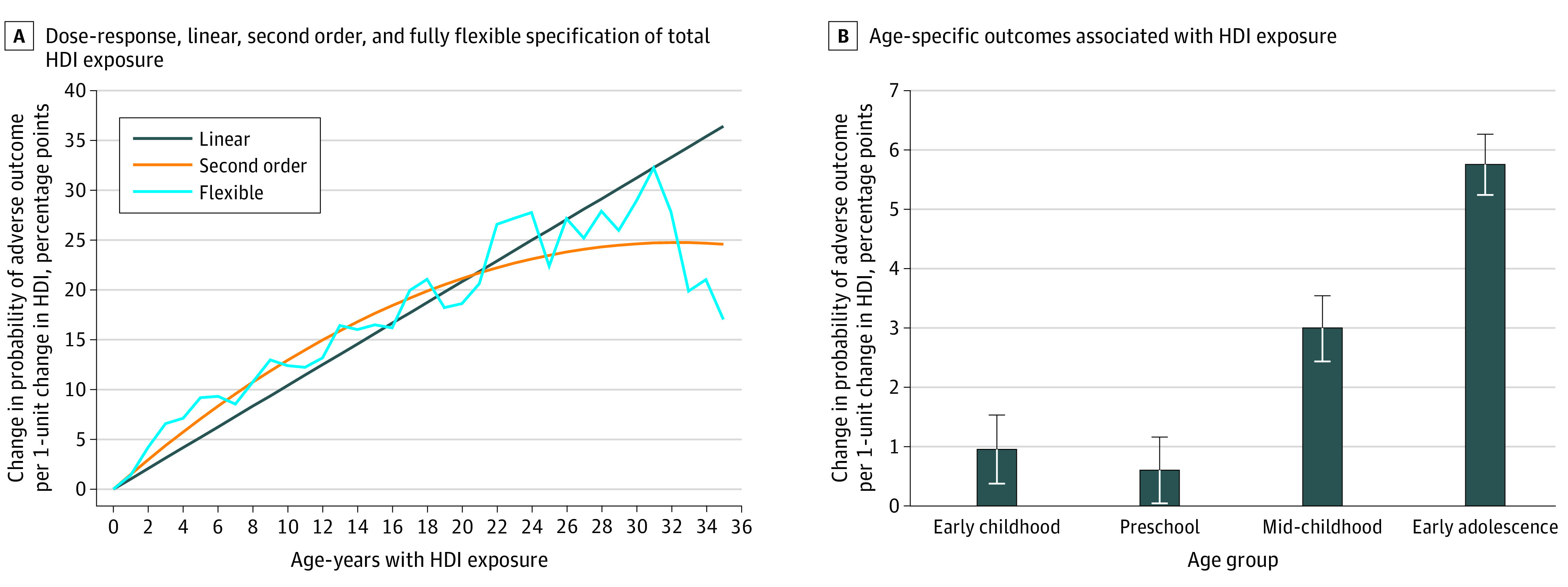
Associations Between Household Dysfunction Items (HDIs) and Later Outcomes A, Standardized regression coefficients were estimated using sibling fixed-effect models (n = 605 344). The model included the total number of age-years with HDI exposure, birth year, birth order, and sex. B, Standardized regression coefficients were estimated using sibling fixed-effect models (n = 605 344). The model included HDI exposure in the 4 age groups, birth year, birth order, and sex. Error bars indicate 95% CIs.

Figure 2A shows the dose-response analysis of the association between the risk of experiencing adverse outcomes and HDI exposure across specification of the HDI measure (linear, second order, or fully flexible function). The analysis revealed a dose-response association of negative childhood experiences with adverse outcomes in adulthood. The standardized regression coefficients from the linear specification suggest that the risk of experiencing the adverse outcomes increased by 1.0 percentage point for each additional HDI that the individual experienced by 17 years of age (β = 0.011; 95% CI, 0.010-0.012; *P* < .001). Results were robust to a daily, rather than a yearly, definition of HDI exposure (eFigure 4 in the Supplement).

Figure 2B shows the age-specific association between HDIs and the adverse outcomes. Exposure in early childhood was associated with an increased risk of experiencing adverse outcomes of 1.0 percentage point (β = 0.010; 95% CI, 0.004-0.015; *P* = .001), and exposure in early adolescence was associated with an increased risk of 5.8 percentage points (β = 0.058; 95% CI, 0.052-0.063; *P* < .001). Compared with the baseline risk of 13.4%, the risk of experiencing an adverse outcome increased 7.4% in association with early childhood exposure and 43.3% in association with adolescent exposure. Except for 1 case, the estimated coefficients were significantly different in the pairwise comparison of age groups (eTable 4 in the Supplement). eFigure 5 in the Supplement shows results by each outcome.

**Figure 3.**
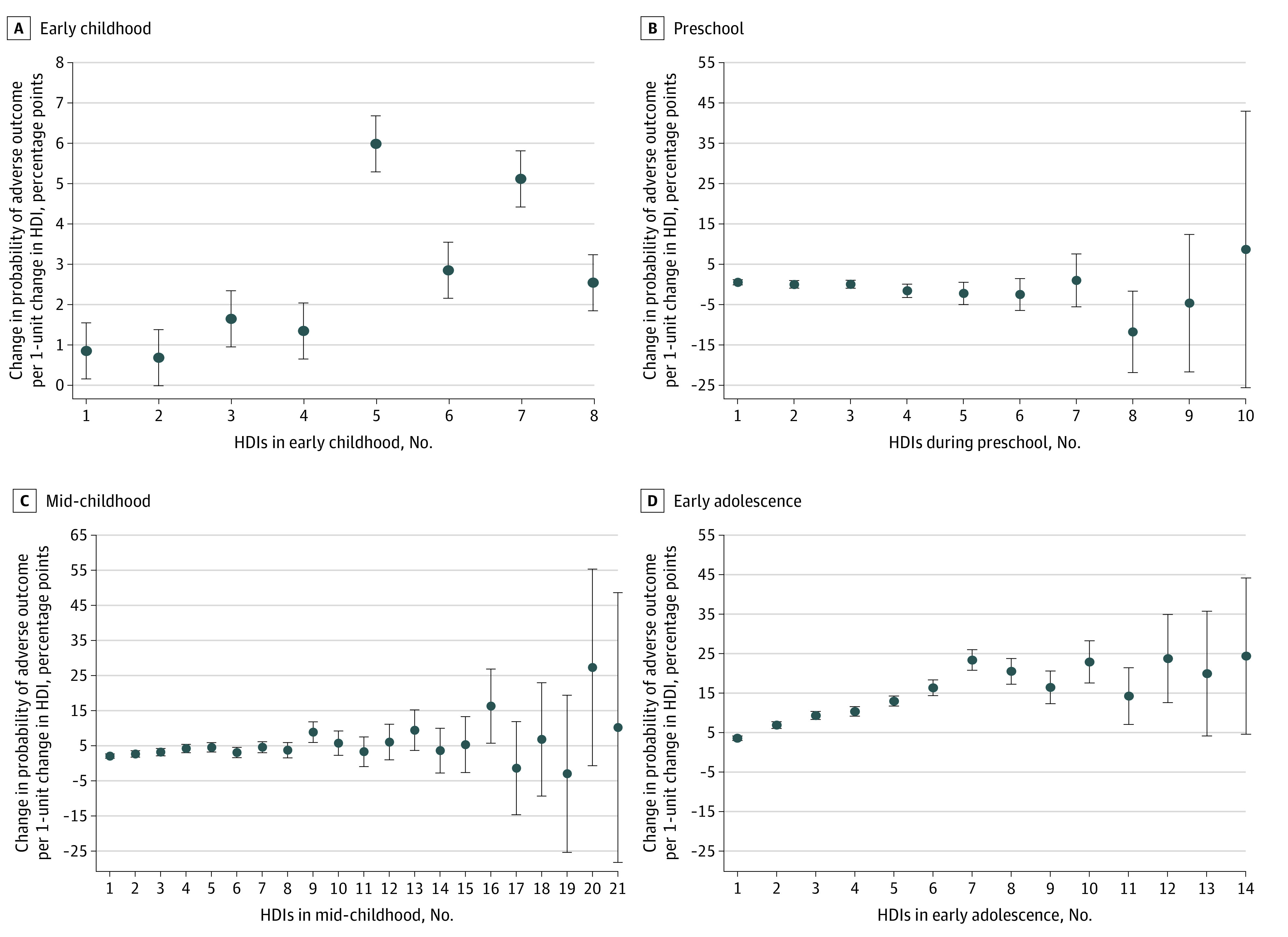
Dose-Response Association Between Household Dysfunction Items and the Risk of Experiencing an Adverse Outcome by Age Category Standardized regression coefficients were estimated from sibling fixed-effect models (n = 605 344). The models include the numbers of age-years with HDI exposure within the 4 age groups, birth year, birth order, and sex. Error bars indicate 95% CIs.

Figure 2 shows the dose-response and age-at-exposure analyses. If HDIs accumulate as a child ages, the dose-response association may confound the age-at-exposure perspective, in which case there would be a dose-response association within the 4 age groups. Figure 3 shows flexible specification of HDI exposure within age groups. The dose-response association reappeared in early adolescence at least until a child had been exposed to 7 HDIs. Exposure to a single HDI was associated with an increase of 3.5 percentage points in the risk of experiencing an adverse outcome (β = 0.035; 95% CI, 0.030-0.041; *P* < .001), but exposure to 7 HDIs was associated with an increased risk of 23.4 percentage points (β = 0.234; 95% CI, 0.176-0.283; *P* < .001). However, fewer indications of a dose-response association were found within the other age groups.

### Proximity of Exposure

**Figure 4.**
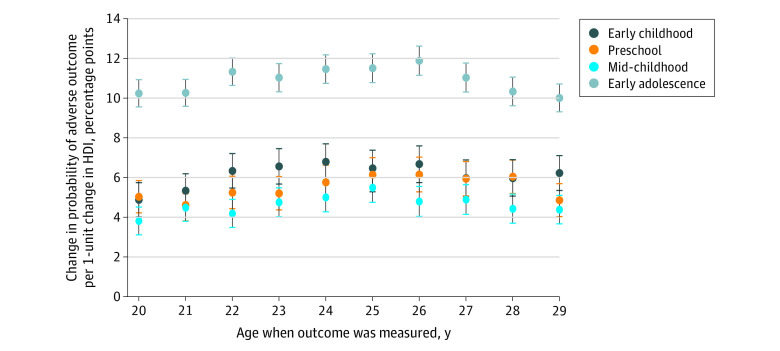
Time Between Household Dysfunction Item (HDI) Exposure and the Outcome in Focus Outcomes were measured between the ages of 20 years and 29 years. Standardized regression coefficients were estimated using ordinary least squares (n = 59 283). The model included HDI exposure in the 4 age groups, birth year, birth order, and sex. Error bars indicate 95% CIs.

The stronger association between HDI exposure in early adolescence and outcomes in early adulthood compared with HDI exposure in childhood may reflect time between exposure and when the outcome was observed. In this case, associations would decrease in size when the time between exposure and outcome was increased. I tested this theory by modeling the association between exposure in each of the 4 age groups and the risk of experiencing adverse outcomes, measured at each age from 20 years to 29 years. There was a stronger association between exposure in early adolescence and the outcome when measured at age 29 years (β = 0.100; 95% CI, 0.093-0.107; *P* < .001) compared with exposure in early childhood and outcomes at 20 years of age (β = 0.049; 95% CI, 0.040-0.047; *P* < .001) (Figure 4). A similar result was found when comparing associations between exposure in early adolescence and the outcome measured at age 29 years with associations between exposure in mid-childhood and the outcome at age 20 years (β = 0.038; 95% CI, 0.031-0.045; *P* < .001), where distance in time between exposure and when the outcome is measured is smallest for the second association (8-14 years vs 12-16 years). This finding suggests that proximity does not drive the age-specific pattern presented in Figure 2.

### Association of Age at HDI Exposure and Outcomes by Maternal Age

Parents’ age confounds child’s age, and the age-specific associations between the HDIs and the adverse outcomes may also reflect variation in parental problems by parental age. I analyzed whether the age-specific associations found in Figure 2 masked the influence of the parents’ age at the time that the HDI occurred (eFigure 7 in the Supplement). The figure supports the findings in Figure 2, with the largest association seen between the outcome and HDI exposure in early adolescence. The coefficient of HDI exposure in early adolescence was statistically different from the other coefficients in all samples (eTable 5 in the Supplement gives the *F* statistics of differences between coefficients), supporting the finding that exposure at this age differed from exposure at other ages. Few of the age-specific associations between HDI exposure and later adverse outcomes differed between subsamples, suggesting that the age-specific associations between HDI exposure and later adverse outcomes did not vary by maternal age.

### Association of Age at HDI Exposure and Outcomes by Type of HDI

**Figure 5.**
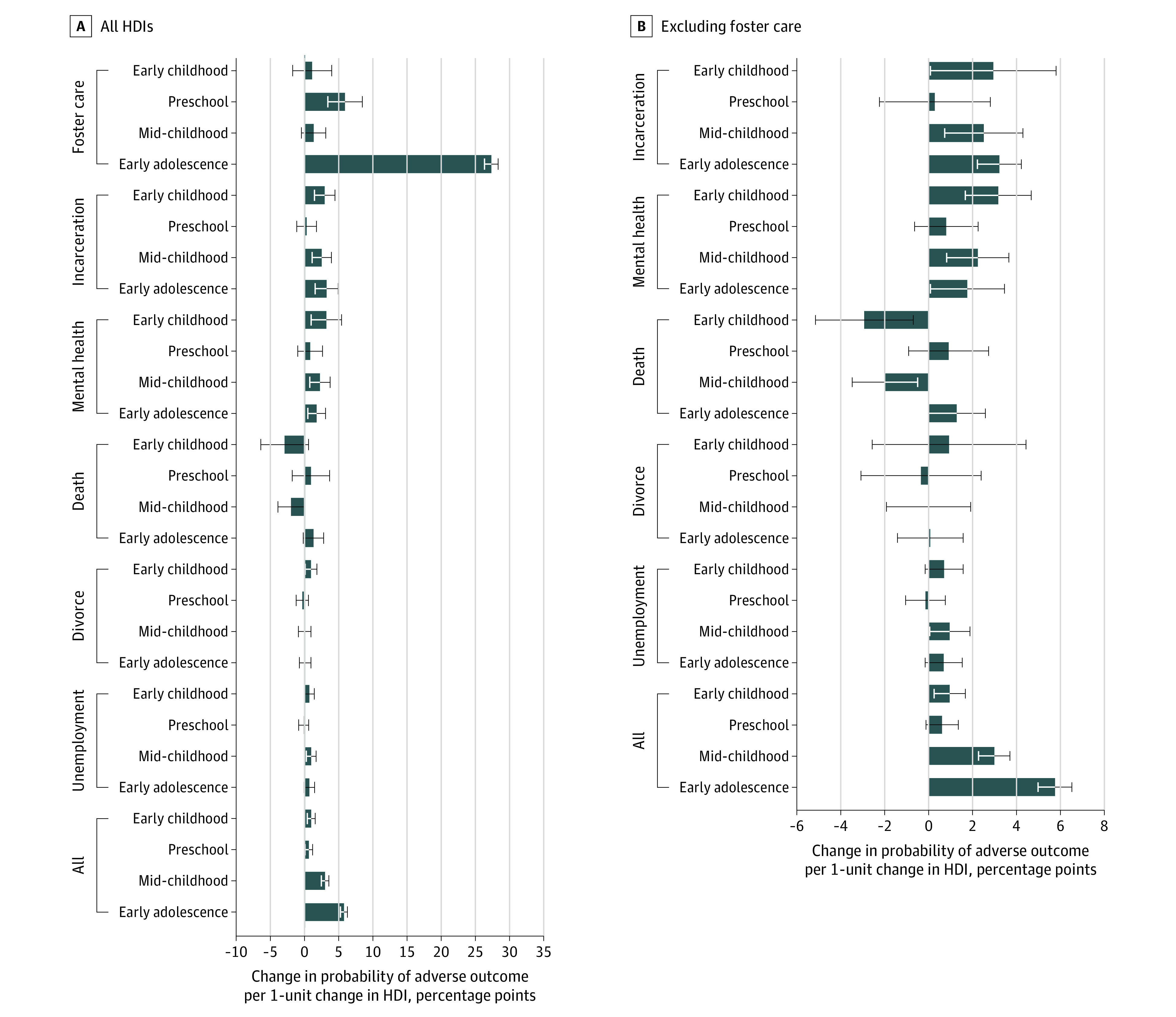
Association Between Household Dysfunction Item (HDI) Exposure and Adverse Outcome by Type of HDI Standardized regression coefficients were estimated from sibling fixed-effect models (n = 605 344). For comparison, the figure also shows the collated HDI measure. The models included HDI exposure in the 4 age groups, birth year, birth order, and sex. Error bars indicate 95% CIs.

Figure 5 shows the association of age at HDI with outcomes exposure by type of HDI.^10,11^ Exposure to incarceration of the father (β = 0.030; 95% CI, 0.014-0.044; *P* < .001), mental illness treatment (β = 0.032; 95% CI, 0.009-0.054; *P* = .005), and parental divorce in early childhood was associated with adverse outcomes (β = 0.009; 95% CI, 0.001-0.018; *P* = .03). Furthermore, exposure to foster care (β = 0.273; 95% CI, 0.263-0.283; *P* < .001), incarceration of the father (β = 0.032; 95% CI, 0.015-0.049; *P* < .001), and mental illness treatment (β = 0.018; 95% CI, 0.005-0.031; *P* = .008) in early adolescence was associated with adverse outcomes. In addition, exposure to parental unemployment (β = 0.010; 95% CI, 0.002-0.017; *P* = .009), incarceration (β = 0.025; 95% CI, 0.011-0.039; *P* = .001), mental illness treatment (β = 0.022; 95% CI, 0.008-0.037; *P* = .003), and parental death (β = −0.020; 95% CI, −0.039 to 0.001; *P* = .04) in mid-childhood was associated with adverse outcomes. The estimated coefficients are shown in eTable 6 in the Supplement.

I examined whether the association between foster care in early adolescence and adverse outcomes influenced the overall results. Although foster care was correlated with the other HDIs, these correlations were too small to explain associations found between the other HDIs and the adverse outcomes (correlations varied between −0.0043 and 0.1637) (eTable 2 in the Supplement). An HDI measure that excluded foster care was used to test whether the association between foster care and adverse outcomes influenced the overall results (eFigure 6 in the Supplement). The estimated coefficients of exposure during early adolescence and early childhood were similar and not significantly different (β = 0.005 [95% CI, 0.003-0.008; *P* < .001] vs β = 0.005 [95% CI, 0.002-0.009; *P* = .001]). The estimated coefficient of exposure during early adolescence was significantly different from the other coefficients (eTable 7 in the Supplement shows test statistics), supporting the finding that age at exposure to HDIs was associated with later risk of experiencing adverse outcomes.

## Discussion

This cohort study yielded 3 insights. First, I found age-specific associations between HDIs and later adverse outcomes, yet I also found a dose-response association between HDIs experienced in early adolescence and later adverse outcomes, suggesting that the dose-response association was contingent on age at exposure. This finding may reflect the sensitivity of the adolescent brain or suggests that activities disrupted by HDIs during adolescence (eg, education) are more vital for later outcomes than the activities disrupted during early childhood. Second, the age-specific associations did not seem to reflect proximity in time between exposure and the outcome measure. Third, not all HDIs had significant associations with outcomes overall or within each age group; foster care in particular was associated with later adverse outcomes.

These insights have several implications. First, the age-specific associations between HDIs and later outcomes resonate with anecdotal evidence about exposure to negative experiences, but my study is among the first to enrich this evidence with data that are sufficient to show reliable patterns at the population level. The results presented here represent information for decision-makers seeking to optimize societies’ scarce resources by targeting disadvantaged groups at the right time.

Second, the association between exposure in adolescence and adverse outcomes resonates with the UNICEF initiative^7^ described above, which promotes adolescence as a second window of opportunity for development of appropriate interventions. If individuals are particularly susceptible to negative influences during early adolescence, they may also be likely to be susceptible to positive influences. This knowledge is helpful for understanding the potential for positive developments among adolescents, even among those with heavy problem loads.

Third, the finding that foster care was among the HDIs with the strongest association with adverse outcomes may not be surprising given the existing literature on adverse childhood experiences.^10^ The literature has mainly focused on abuse and neglect, which are often the main reasons for placing a child in foster care. However, from a policy perspective, being able to link these 2 subjective concepts (abuse and neglect) that may be difficult to observe in a child to a tangible system may provide useful information regarding which individuals to target with interventions.

### Limitations

This study has limitations. First, the results reflect a single geographic location and a specific historical period. Denmark was a useful setting because of the country’s generous welfare state, which may ameliorate the consequences of HDIs, and findings from Denmark suggest that there may be stronger associations and larger differences between age-specific exposure than in countries in which consequences of HDIs are not likely to be ameliorated. In particular, the small association found between HDI exposure in early childhood and adverse outcomes may reflect the welfare services provided in Denmark during the first years of life, which are specifically meant to equalize opportunities for growth and development among children. The findings presented here may therefore represent a lower-bound estimate of the associations. Still, replication in other contexts is needed to gain a deeper understanding of age-specific associations of HDIs. Second, although the administrative data provide precise measures of HDI exposure, their objective character reveals little about how an individual experiences the exposure. For example, in the analyses, I define parents’ hospitalization for treatment of mental illness as an HDI; however, in a family in which a parent experiences mental illness, a problem that may be correlated with poor parenting skills, the hospitalization may represent a positive break from stressful parent-child interactions. The actual consequences of this HDI may therefore be the opposite of what I expected.

## Conclusions

In this cohort study, youth age at exposure to 1 or more HDIs was associated with outcomes in early adulthood. Exposure during early adolescence was more strongly associated with the risk of experiencing an adverse outcome compared with earlier points in childhood. The results of the study also suggest a dose-response association between HDIs and later adverse outcomes, yet this finding seems to be most pronounced in early adolescence. An association of proximity between exposure and the time at which the outcome was measured did not influence the overall results. In contrast, foster care was one of the HDIs with the strongest association with adverse outcomes, but the exclusion of this item did not undermine the results.

## Notes

Supplement. **eMethods.** Registers used for identifying the HDIs and the sibling fixed effects model **eTable 1.** Exposure by age, by sample **eTable 2.** Pearson’s correlation coefficients between exposure to the 6 HDIs **eTable 3.** Descriptive statistics **eTable 4.** *F* test for difference between coefficients **eTable 5.** Differences between coefficients within and between models **eTable 6.** Standardized regression coefficients by HDI **eTable 7.** *F* test for difference between coefficients, excluding foster care **eFigure 1.** Total no. of HDIs, cohorts 1987-1995 **eFigure 2.** Share with each HDI **eFigure 3.** Share with adverse outcome measured at age 20-29, reduced sample, cohort 1987 **eFigure 4.** Dose-response relationship **eFigure 5.** Exposure in each age range, by each outcome **eFigure 6.** Exposure in each age range, excluding foster care **eFigure 7.** Age-specific associations, by subgroups defined by mother’s age at childbirth
