# Errors in the Results, Figures 2 to 5, and the Supplement

**DOI:** 10.1001/jamanetworkopen.2021.15032

**Published:** 2021-05-25

In the Original Investigation titled “Association of Youth Age at Exposure to Household Dysfunction With Outcomes in Early Adulthood,”^1^ published January 7, 2021, in the Results section, the captions of Figures 2 to 5, and eTable 6 and eFigures 5 and 6 in the Supplement, “standardized regression coefficients” was changed to “regression coefficients.” In eTable 3 in the Supplement, the definition of “not graduated from primary school” was changed from less than 12 years of education to less than 10 years of education. This article has been corrected.^1^
