# Supplementary material for: Association of Youth Age at Exposure to Household Dysfunction With Outcomes in Early Adulthood
Source: JAMA Netw Open. 2021 Jan 7;4(1):e2032769. doi: 10.1001/jamanetworkopen.2020.32769 (PMC7791359; doi:10.1001/jamanetworkopen.2020.32769)
Supplement: Supplement. — eMethods. Registers used for identifying the HDIs and the sibling fixed effects model eTable 1. Exposure by age, by sample eTable 2. Pearson’s correlation coefficients between exposure to the 6 HDIs eTable 3. Descriptive statistics eTable 4. F test for difference between coefficients eTable 5. Differences between coefficients within and between models eTable 6. Standardized regression coefficients by HDI eTable 7. F test for difference between coefficients, excluding foster care eFigure 1. Total no. of HDIs, cohorts 1987-1995 eFigure 2. Share with each HDI eFigure 3. Share with adverse outcome measured at age 20-29, reduced sample, cohort 1987 eFigure 4. Dose-response relationship eFigure 5. Exposure in each age range, by each outcome eFigure 6. Exposure in each age range, excluding foster care eFigure 7. Age-specific associations, by subgroups defined by mother’s age at childbirth [file jamanetwopen-e2032769-s001.pdf]

Supplemental Online Content

Andersen SH. Association of youth age at exposure to household dysfunction with outcomes in early adulthood. *JAMA Netw Open*. 2021;4(1):e2032769.
doi:10.1001/jamanetworkopen.2020.32769

eMethods. Registers used for identifying the HDIs and the sibling fixed effects model
eTable 1. Exposure by age, by sample
eTable 2. Pearson's correlation coefficients between exposure to the 6 HDIs
eTable 3. Descriptive statistics
eTable 4. *F* test for difference between coefficients
eTable 5. Differences between coefficients within and between models
eTable 6. Standardized regression coefficients by HDI
eTable 7. *F* test for difference between coefficients, excluding foster care
eFigure 1. Total no. of HDIs, cohorts 1987-1995
eFigure 2. Share with each HDI
eFigure 3. Share with adverse outcome measured at age 20-29, reduced sample, cohort 1987
eFigure 4. Dose-response relationship
eFigure 5. Exposure in each age range, by each outcome
eFigure 6. Exposure in each age range, excluding foster care
eFigure 7. Age-specific associations, by subgroups defined by mother's age at childbirth

This supplemental material has been provided by the authors to give readers additional information about their work.

© 2021 Andersen SH. *JAMA Network Open.*

eMethods:

Registers used for identifying the HDIs:

1. Parental divorce: I identified parental divorce using the date parents move to separate addresses, which is available from the register BEF.
2. Parents' prolonged unemployment (>9 months within a year): I identified parents prolonged unemployment using start and end weeks on take-up of welfare benefits, that is available from the registers DREAM/SHSS).
3. Father's incarceration: I identified fathers incarceration using the start and end dates of incarceration spells, that is available from the register KRIN. Note that I did not include information on mother's incarceration as only very few women are incarcerated in Denmark
4. Parents' inpatient mental health treatment: I Identified parents' inpatient mental health treatment using start and end dates of inpatient stays at mental health hospitals, that is available from the register LPR_PSYK.
5. The child's foster care placement: I identified the child's foster care placement using the start and end dates of stays in a residential care/group home or at a foster family, that is available from the register BUA.
6. Parents' death: I identified parents' death using information on date of death that is available from the register DOD.

The Sibling fixed effects model: The SFE adjusts for observed and unobserved confounders that siblings share (e.g. half their genes and time constant environmental influences), and which may bias the estimate. For this purpose, the model relies on variation between siblings, and thus only on sibling groups consisting of 2 or more individuals. Equation 1 shows the SFE, when estimated using the four age-band HDI-indicators:

$$\Delta Y_{if} = \beta_1 \Delta HDI_{if1} + \beta_2 \Delta HDI_{if2} + \beta_3 \Delta HDI_{if3} + \beta_4 \Delta HDI_{if4} + \delta \Delta X_{if} + \alpha_f + \Delta \varepsilon_{if}$$

In the model I regressed the outcome Y, for individual *i* in family *f* on HDI exposure in early childhood (HDI_1), preschool years (HDI_2), mid-childhood (HDI_3) and early adolescence (HDI_4). The identification of coefficients β_1- β_4 rests on within-family sibling differences on HDI_1- HDI_4. I defined siblings as individuals with the same mother and of the 605,344 individuals in my sample, 363,444 are nested in sibling groups with 2 to 8 individuals. The average age difference in sibling groups is between 3.0 years (sibling groups with 2 individuals) and 7.5 years (sibling groups with 8 individuals), age differences large enough to facilitate between-sibling variation in age at HDI exposure. Additional calculations showed that variation between 45,193 siblings contributed to the estimation of HDI_1, 49,554 contributed to the estimation of HDI_2, 70,374 to the estimation of HDI_3 and 61,726 to the estimation of HDI_4. The vector of controls, X, includes birth order, birth year and child gender for the individual *i* in family *f*. I estimated the models using the statistical software package StataMP15

© 2021 Andersen SH. *JAMA Network Open.*

eTable 1: Share/cumulative share (in percentages) with HDI Exposure by age, by sample. Standard deviation in parentheses

	Full sample		Reduced sample		Sample A Mothers<25 at child's birth		Sample B: 25<=Mothers<30 at child's birth		Sample C: 30<=Mothers<35 at child's birth		Sample D: 35<=Mothers<40 at child's birth	
Age	Share	Cum. share	Share	Cum. share	Share	Cum. share	Share	Cum. share	Share	Cum. share	Share	Cum. share
0	8.5 (0.279)	8.5	6.6 (0.248)	6.6	18.2 (0.386)	29.3	6.7 (0.249)	6.7	5.2 (0.222)	5.2	5.9 (0.235)	5.9
1	11.3 (0.317)	13.3	9.6 (0.294)	11.2	24.9 (0.432)	28.1	8.8 (0.284)	10.5	6.7 (0.250)	8.1	7.5 (0.263)	9.0
2	11.3 (0.317)	16.7	11.1 (0.314)	15.5	23.5 (0.424)	33.6	9.1 (0.288)	13.9	7.0 (0.256)	10.7	7.8 (0.268)	11.6
3	11.3 (0.316)	19.7	11.1 (0.314)	18.8	22.8 (0.419)	37.7	9.3 (0.290)	16.8	7.1 (0.258)	13.0	8.0 (0.271)	14.0
4	11.1 (0.315)	22.2	11.5 (0.319)	21.9	22.0 (0.415)	41.1	9.2 (0.288)	19.4	7.4 (0.261)	15.2	7.9 (0.270)	16.0
5	11.0 (0.313)	24.6	12.2 (0.327)	24.8	21.4 (0.410)	44.0	9.1 (0.288)	21.8	7.5 (0.263)	17.4	8.2 (0.274)	18.0
6	10.8 (0.310)	26.9	12.4 (0.330)	27.4	20.5 (0.404)	46.5	9.1 (0.287	24.2	7.4 (0.261)	19.4	8.1 (0.272)	20.0
7	10.6 (0.308)	29.0	12.4 (0.329)	29.9	19.9 (0.399)	48.8	8.9 (0.285)	26.3	7.3 (0.260)	21.3	8.1 (0.272)	22.0
8	10.4 (0.305)	31.0	11.1 (0.315)	31.8	19.3 (0.394)	50.9	8.7 (0.282)	28.4	7.2 (0.259)	23.1	8.1 (0.273)	23.9
9	10.3 (0.304)	32.9	10.3 (0.303)	33.3	19.0 (0.392)	53.0	8.8 (0.283)	30.5	7.3 (0.260)	25.0	7.9 (0.280)	25.6
10	10.4 (0.305)	34.8	9.6 (0.294)	34.3	18.9 (0.392)	54.9	8.8 (0.282)	32.3	7.3 (0.261)	26.8	8.1 (0.272)	27.4
11	10.4 (0.306)	36.6	10.1 (0.301)	35.7	19.0 (0.392)	56.9	8.9 (0.285)	34.3	7.3 (0.261)	28.5	8.0 (0.271)	29.1
12	10.3 (0.304)	38.4	10.4 (0.305)	37.4	18.8 (0.391)	58.7	8.8 (0.284)	36.2	7.2 (0.258)	33.0	7.9 (0.270)	30.6
13	9.9 (0.299)	41.0	10.9 (0.311)	37.1	18.2 (0.386)	60.3	8.5 (0.279)	37.9	7.0 (0.255)	31.9	7.6 (0.265)	32.1
14	9.3 (0.291)	41.6	10.9 (0.311)	40.7	17.0 (0.375)	61.8	8.0 (0.271)	39.5	6.6 (0.248)	33.4	7.1 (0.257)	33.6
15	9.0 (0.286)	43.2	11.3 (0.317)	42.7	16.3 (0.369)	63.2	7.7 (0.266)	41.1	6.4 (0.244)	34.9	6.9 (0.254)	35.1
16	8.6 (0.280)	44.6	11.6 (0.320)	44.3	15.4 (0.360)	64.5	7.3 (0.261)	42.7	6.1 (0.240)	36.3	6.7 (0.250)	36.5
17	8.2 (0.274)	45.9	11.7 (0.322)	45.8	14.4 (0.351)	65.7	7.0 (0.255)	44.0	5.9 (0.235)	37.7	6.8 (0.251)	37.9
N	605,344		59,283		123,096		240,289		171,765		70,194	

© 2021 Andersen SH. *JAMA Network Open.*

eTable 2: Pearson's correlation coefficients between exposure to the 6 HDIs (N=605,344)

a: Exposure in early childhood

	Unemploy-ment	Foster care	Incarcera-tion	Parents' men. health	Parental death	Divorce
Unemployment		0.1006	0.1703	0.0792	0.0279	0.1722
Foster care	0.1006		0.0742	0.1521	0.0416	0.0468
Incarceration	0.1703	0.0742		0.0566	0.0257	0.0847
Parents' mental health	0.0792	0.1521	0.0566		0.0425	0.0676
Parental death	0.0279	0.0416	0.0257	0.0425		0.0427
Divorce	0.1722	0.0468	0.0847	0.0676	0.0427	

b: Exposure during preschool years

	Unemploy-ment	Foster care	Incarcera-tion	Parents' men. health	Parental death	Divorce
Unemployment		0.1298	0.1883	0.0971	0.0342	0.0907
Foster care	0.1298		0.0993	0.1451	0.0587	0.0259
Incarceration	0.1883	0.0993		0.0687	0.0313	0.0427
Parents' mental health	0.0971	0.1451	0.0687		0.0529	0.0544
Parental death	0.0342	0.0587	0.0313	0.0529		0.0324
Divorce	0.0907	0.0259	0.0427	0.0544	0.0324	

c: Exposure during mid-childhood

	Unemploy-ment	Foster care	Incarcera-tion	Parents' men. health	Parental death	Divorce
Unemployment		0.1614	0.2152	0.1395	0.0475	0.0415
Foster care	0.1614		0.1347	0.1637	0.0832	0.0121
Incarceration	0.2152	0.1347		0.1057	0.0424	0.0212
Parents' mental health	0.1395	0.1637	0.1057		0.0729	0.0706
Parental death	0.0475	0.0832	0.0424	0.0729		0.0188
Divorce	0.0415	0.0121	0.0212	0.0706	0.0188	

c: Exposure during early adolescence

	Unemploy-ment	Foster care	Incarcera-tion	Parents' men. health	Parental death	Divorce
Unemployment		0.1583	0.1480	0.1081	0.0358	0.0046
Foster care	0.1583		0.1108	0.1322	0.0764	-0.0043
Incarceration	0.1480	0.1108		0.0832	0.0240	0.0023
Parents' mental health	0.1081	0.1322	0.0832		0.0674	0.0519
Parental death	0.0358	0.0764	0.0240	0.0674		0.0116
Divorce	0.0046	-0.0043	0.0023	0.0519	0.0116	

© 2021 Andersen SH. *JAMA Network Open.*

eTable 3: Descriptive statistics

A: Outcome measures, definition and means(std), by sample

		Full sample	Sample A	Sample B	Sample C	Sample D
Indicator	Definition	Mean (std.)	Mean (std.)	Mean (std.)	Mean (std.)	Mean (std.)
Disconnected youth	No labor market or educational activity at age 18 and 19. Information available from the register RAS (measured in November).	0.033 (0.179)	0.048 (0.214)	0.029 (0.169)	0.028 (0.164)	0.033 (0.179)
Not graduated from primary school	Less than 12 years of education. Information available from the register UDDA	0.020 (0.141)	0.032 (0.177)	0.019 (0.135)	0.016 (0.124)	0.017 (0.128)
Mental health problems	Diagnosed with a mental disorder (ICD-10 code: F00-F99) at a mental health facility at age 18 or 19. Information available from the register LPR_PSYK	0.039 (0.193)	0.051 (0.219)	0.036 (0.187)	0.035 (0.183)	0.037 (0.189)
Criminal offence	Having been charged with a criminal offence at age 18 or 19. Information available from the register KRAF	0.068 (0.252)	0.105 (0.307)	0.063 (0.243)	0.054 (0.227)	0.054 (0.226)
Collated measure	Takes the value 1 (and zero otherwise) if any of the four indicators above (“disconnected youth”, “Not graduated from primary school”, “Mental health problems” and “Criminal offence”) takes the value 1	0.134 (0.341)	0.192 (0.394)	0.124 (0.330)	0.113 (0.317)	0.120 (0.324)
N		605,344	123,096	240,289	171,765	70,194

B: Pearson’s correlation coefficients between outcome measures, full sample (N=605,344)

	Disconnected youth	Not grad. From primary school	Mental health problems	Criminal offence
Disconnected youth		0.1036	0.1857	0.0706
Not graduated from primary school	0.1036		0.1857	0.0570
Mental health problems	0.1857	0.0570		0.0703
Criminal offence	0.0706	0.1064	0.0703	

© 2021 Andersen SH. *JAMA Network Open.*

C: Control variables, mean(std), by sample

	Full sample	Reduced sample	Sample A	Sample B	Sample C	Sample D
Variable	Mean (std.)	Mean (std.)	Mean (std.)	Mean (std.)	Mean (std.)	Mean (std.)
Birth order	1.377 (0.596)	1.011 (0.106)	1.209 (0.464)	1.367 (0.581)	1.490 (0.649)	1.427 (0.640)
Sex (1=female)	0.49 (0.50)	0.480 (500)	0.48 (0.50)	0.49 (0.50)	0.49 (0.50)	0.49 (0.50)
Birth year	1991 (2.563)	1987 (0)	1991 (2.569)	1991 (2.533)	1991 (2.565)	1991 (2.554)
N	605,344	59,283	123,096	240,289	171,765	70,194

eTable 4: *F* test for difference between coefficients (p-values for statistical significance in parentheses), (N=605,344)

	Early childhood	Preschool	Mid childhood	Early adolescence
Early Childhood		0.79 (p=0.375)	25.72 (p=0.000)	146.20 (p=0.000)
Preschool	0.79 (p=0.375)		42.30 (p=0.000)	180.21(p=0.000)
Mid childhood	25.72 (p=0.000)	42.30 (p=0.000)		67.14 (p=0.000)
Early adolescence	146.20 (p=0.000)	180.21 (p=0.000)	67.14 (p=0.000)	

eTable 5: Differences between coefficients within and between models, by sub samples as defined by mother's age at child birth

Panel A: F-test for difference between coefficients (p-values for statistical significance in parentheses)

	Early childhood	Preschool	Mid childhood	Early adolescence
Sample A: Mothers<25 at child's birth (N=123,096)				
Early Childhood		0.00 (p=0.945)	1.51 (p=0.219)	37.86 (p=0.000)
Preschool	0.00 (p=0.945)		1.83 (p=0.177)	38.06 (p=0.000)
Mid childhood	1.51 (p=0.219)	1.83 (p=0.177)		23.73 (p=0.000)
Early adolescence	37.86 (p=0.000)	38.06 (p=0.000)	23.73 (p=0.000)	
Sample B: 25<=Mothers<30 at child's birth (N=240,289)				
Early Childhood		0.14 (p=0.705)	1.69 (p=0.193)	24.28 (p=0.000)
Preschool	0.14 (p=0.705)		3.70 (p=0.054)	30.98 (p=0.000)
Mid childhood	1.69 (p=0.193)	3.70 (p=0.054)		18.90 (p=0.000)
Early adolescence	24.28 (p=0.000)	30.98 (p=0.000)	18.90 (p=0.000)	
Sample C: 30<=Mothers<35 at child's birth (N=171,765)				
Early Childhood		0.18 (p=0.673)	13.86 (p=0.000)	25.89 (p=0.000)
Preschool	0.18 (p=0.673)		22.28 (p=0.000)	33.58 (p=0.000)
Mid childhood	13.86 (p=0.000)	22.28 (p=0.000)		2.44 (p=0.118)
Early adolescence	25.89 (p=0.000)	33.58 (p=0.000)	2.44 (p=0.118)	
Sample D: 35<=Mothers<40 at child's birth (N=70,194)				
Early Childhood		0.06 (p=0.805)	1.35 (p=0.245)	4.25 (p=0.039)
Preschool	0.06 (p=0.805)		2.42 (p=0.120)	5.71 (p=0.017)
Mid childhood	1.35 (p=0.245)	2.42 (p=0.120)		0.98 (p=0.321)
Early adolescence	4.25 (p=0.039)	5.71 (p=0.017)	0.98 (p=0.321)	

© 2021 Andersen SH. *JAMA Network Open.*

Panel B:Test for differences in coefficients, confidence intervals in squared parentheses, t-test-statistics

Age at exposure	Samples being compared		Diff [conf. Intervals]	t-test
Early childhood	A	B	-0.011 [-0.034; 0.013]	-0.872
	A	C	-0.007 [-0.036; 0.021]	-0.498
	A	D	-0.002 [-0.036; 0.031]	-0.145
	B	C	0.003 [-0.022; 0.028]	0.250
	B	D	0.008 [-0.025; 0.041]	0.476
	C	D	0.005 [-0.035; 0.045]	0.235
Preschool	A	B	-0.008 [-0.031; 0.015]	-0.664
	A	C	-0.003 [-0.031; 0.026]	-0.174
	A	D	0.002 [-0.031; 0.036]	0.129
	B	C	0.005 [-0.019; 0.029]	0.431
	B	D	0.010 [-0.022; 0.042]	0.618
	C	D	0.005 [-0.034; 0.043]	0.239
Mid-childhood	A	B	-0.008 [-0.031; 0.015]	-0.659
	A	C	-0.045 [-0.073; -0.017]	-3.184
	A	D	-0.014 [-0.048; 0.020]	-0.805
	B	C	-0.037 [-0.061; -0.014]	-3.160
	B	D	-0.006 [-0.037. 0.025]	-0.401
	C	D	0.031 [-0.006; 0.068]	1.648
Early adolescence	A	B	0.016 [-0.005; 0.037]	1.490
	A	C	-0.004 [-0.028; 0.029]	-0.302
	A	D	0.026 [-0.004; 0.057]	1.681
	B	C	-0.020 [-0.040; 0.001]	-1.891
	B	D	0.010 [-0.017; 0.037]	0.732
	C	D	0.030 [-0.002; 0.062]	1.838

eTable 6: Standardized regression coefficients (95% CI in squared parentheses), by HDI, (N=605,344)

	Unemploy-ment	Foster care	Incarceration	Parents' men. health	Parental death	Divorce
Early Childh.	.007 [-.000;.014]	.011 [-.018;.040]	.029 [.014;.044]	.032 [.009;.054]	-.029 [-.064;.006]	.009 [.001;.018]
Preschool	-.001 [-.009;.006]	.059 [.034;.084]	.003 [-.012;.017]	.008 [-.010;.026]	.009 [-.018;.036]	-.003 [-.013;.009]
Mid childh	.010 [.002;.017]	.013 [-.005;.031]	.025 [.011;.039]	.022 [.008;.037]	-.020 [-.039;-.001]	.000 [-.009;.009]
Early adoles.	.007 [-.001;.015]	.273 [.263;.283]	.032 [.015;.049]	.018 [.005;.031]	.013 [-.002;.028]	.001 [-.008;.009]

© 2021 Andersen SH. *JAMA Network Open.*

eTable 7: *F* test for difference between coefficients, excluding foster care (p-values for statistical significance in parentheses), (N=605,344)

	Early childhood	Preschool	Mid childhood	Early adolescence
Early Childhood		7.10 (p=0.008)	2.73(p=0.098)	0.01 (p=0.919)
Preschool	7.10 (p=0.008)		2.85 (p=0.092)	8.99 (p=0,003)
Mid childhood	2.73 (p=0.098)	2.85 (p=0.092)		4.01 (p=0.045)
Early adolescence	0.01 (p=0.919)	8.99 (p=0.003)	4.01 (p=0.045)	

© 2021 Andersen SH. *JAMA Network Open.*

eFigures

eFigure 1: Total no. of HDIs, cohorts 1987-1995 (N=605,344)

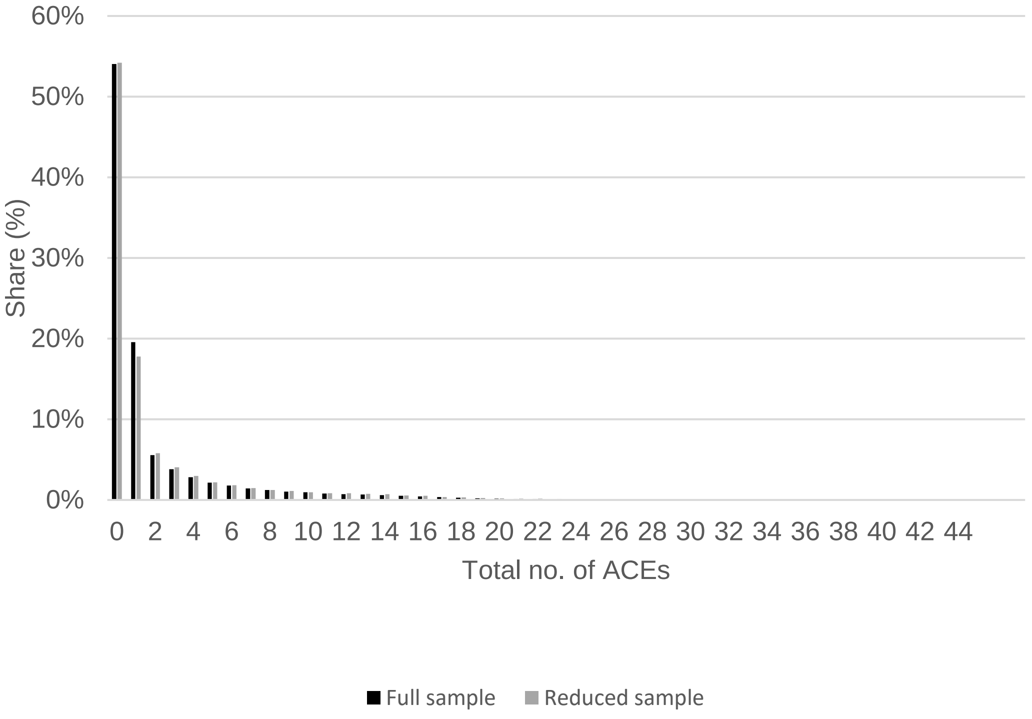

eFigure 2: Share with each HDI

Panel A: By continuous age, full sample, cohorts 1987-1995 (N=605,344)

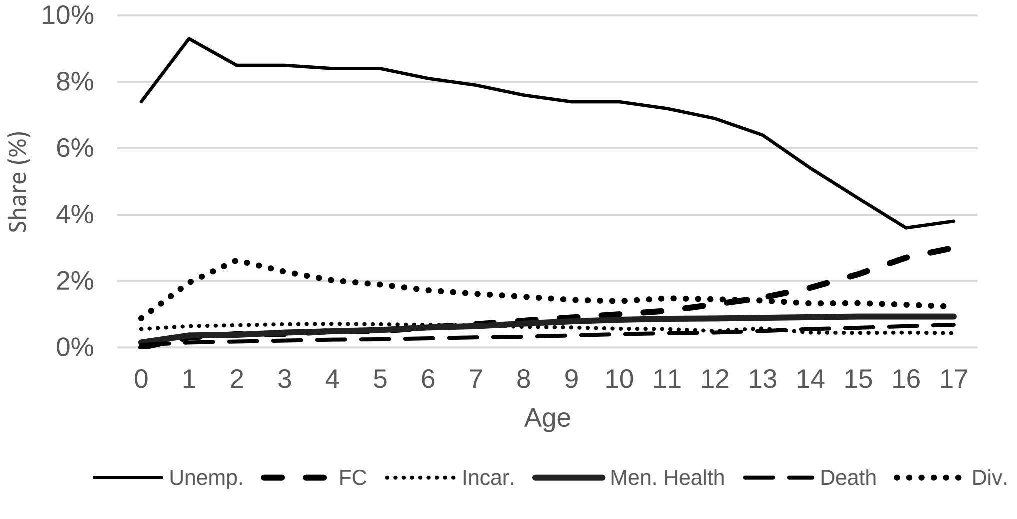

© 2021 Andersen SH. *JAMA Network Open.*

Panel B: By continuous age, reduced sample, 1987 cohort (N=59,283)

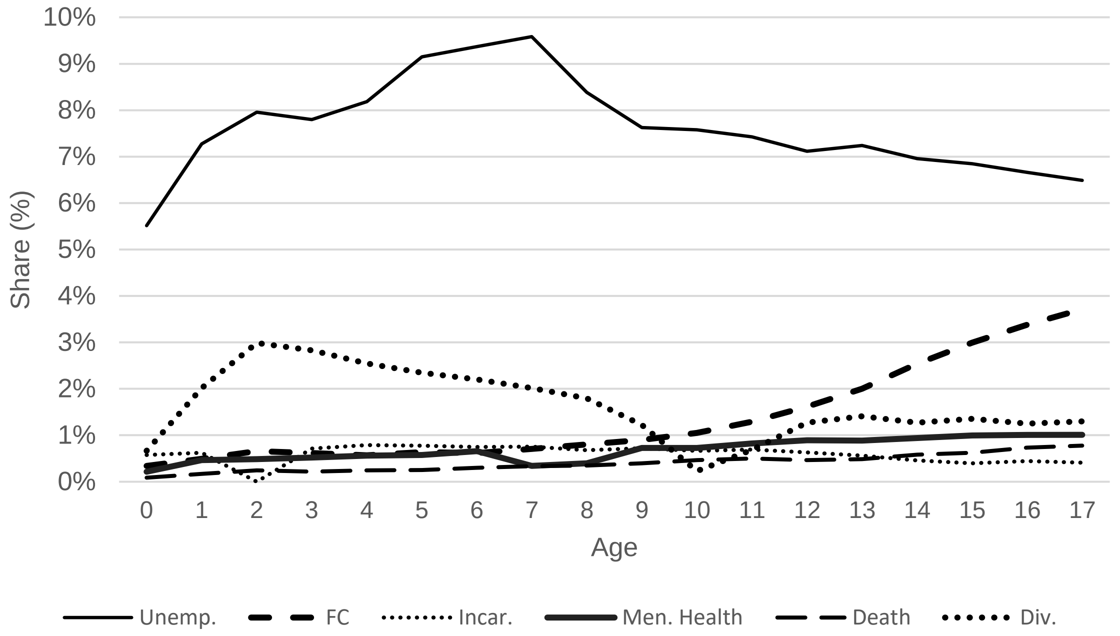

Panel C: By age-band, full sample, cohorts 1987-1995 (N=605,344)

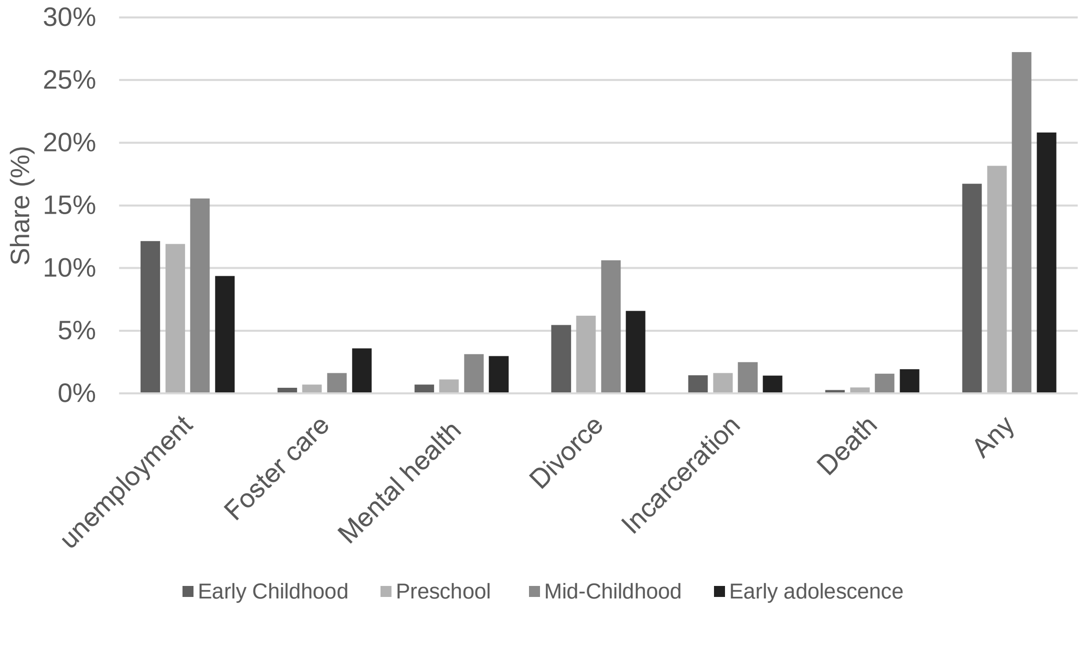

© 2021 Andersen SH. *JAMA Network Open.*

Panel D: By age-band, reduced sample, cohort 1987 (N=59,283)

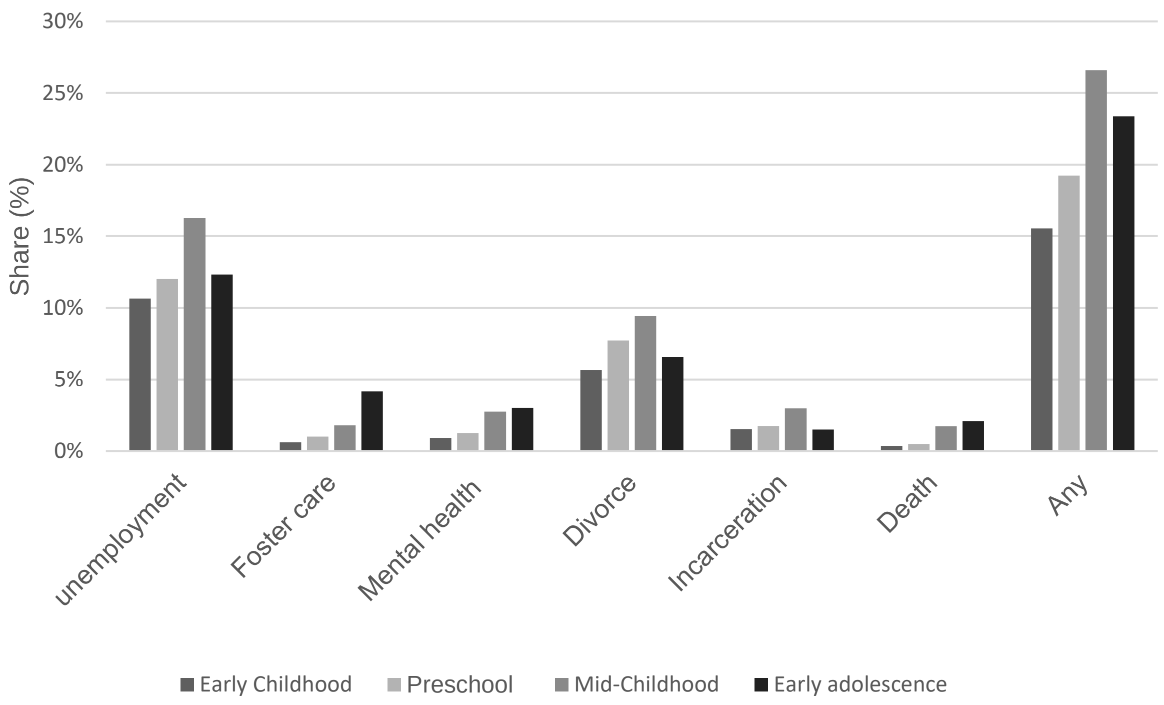

eFigure 3: Share with adverse outcome measured at age 20-29, reduced sample, cohort 1987 (N=59,283)

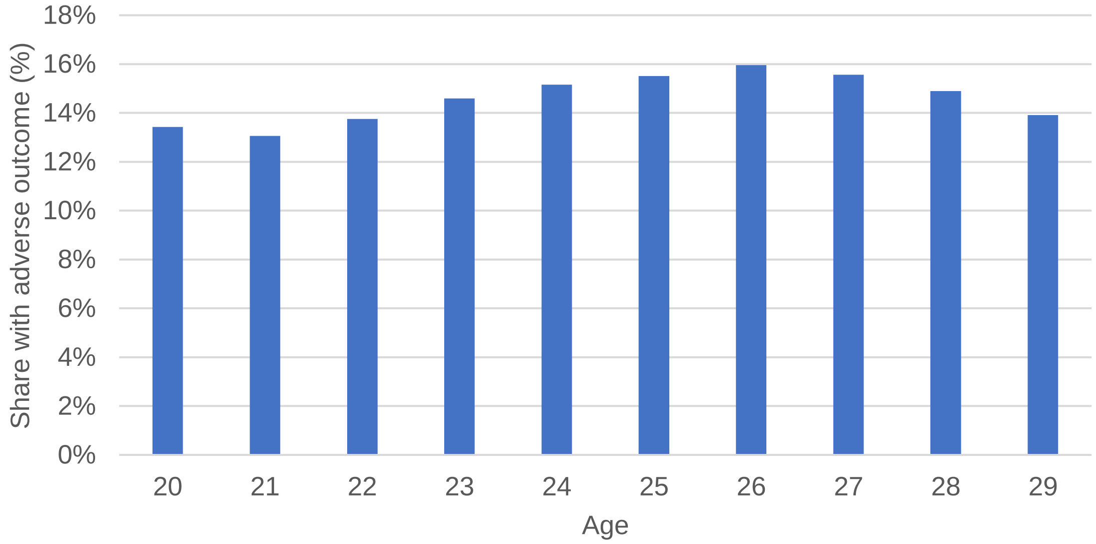

© 2021 Andersen SH. *JAMA Network Open.*

eFigure 4: Dose-response relationship, HDI defined on the basis of daily exposure (top-coded at 10,000 days of HDI exposure, mean days with HDIs=1980.77 [min=0; max=10000]. Estimated using sibling fixed effects models (N=605,344)

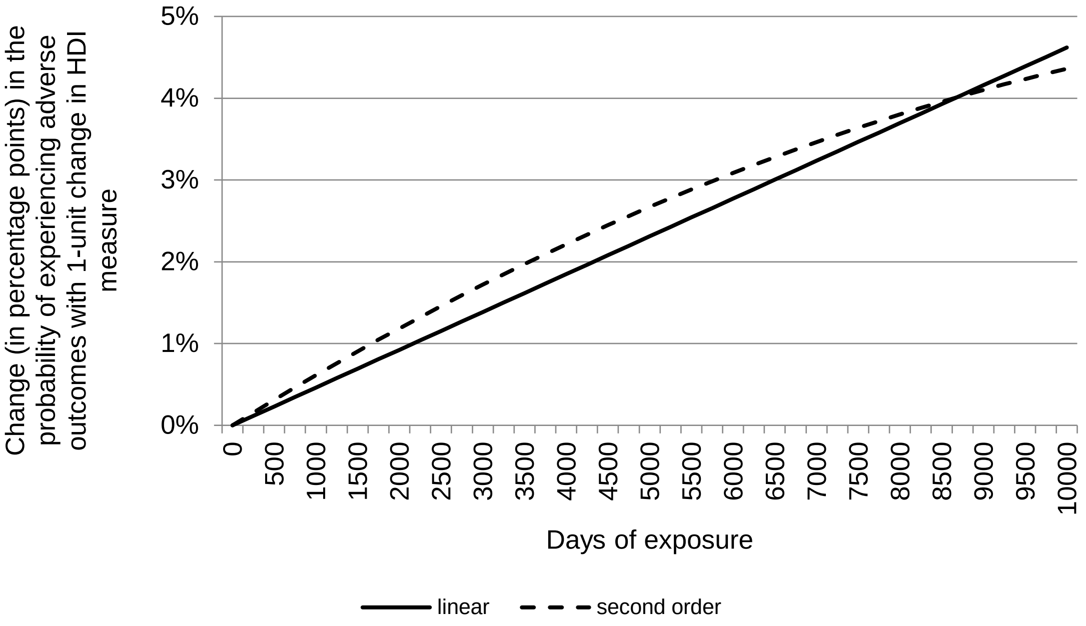

Note: Parental death and divorce is a binary occurrence that does not stretch over time but happen at a specific point in time. To allow these negative events to enter into the measure with full impact, I assign these experiences a value of 365, whereby allowing exposure to these two events to count as much as e.g. fathers 365 days of incarceration, parents 365 days of inpatient treatment at a mental health facility or parents' 365 days of unemployment. Aside from the count indicator of total days of HDI exposure, the model includes birth year, birth order and gender

© 2021 Andersen SH. *JAMA Network Open.*

eFigure 5: Exposure in each age range, by each outcome. Standardized regression coefficients from sibling fixed effects models (N=605,344)

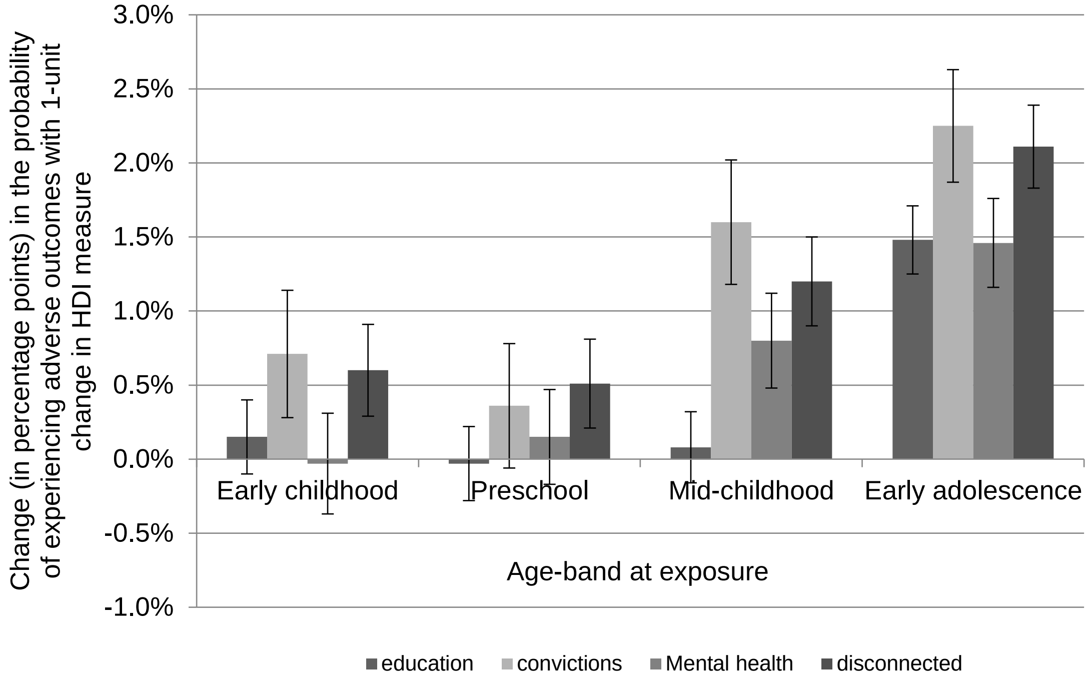

The model includes the indicators of HDI exposure in the four age-bands, birth year, birth order and gender. Error bars indicate 95% CIs

© 2021 Andersen SH. *JAMA Network Open.*

eFigure 6: Exposure in each age range, excluding foster care. Standardized regression coefficients from sibling fixed effects models (N=605,344)

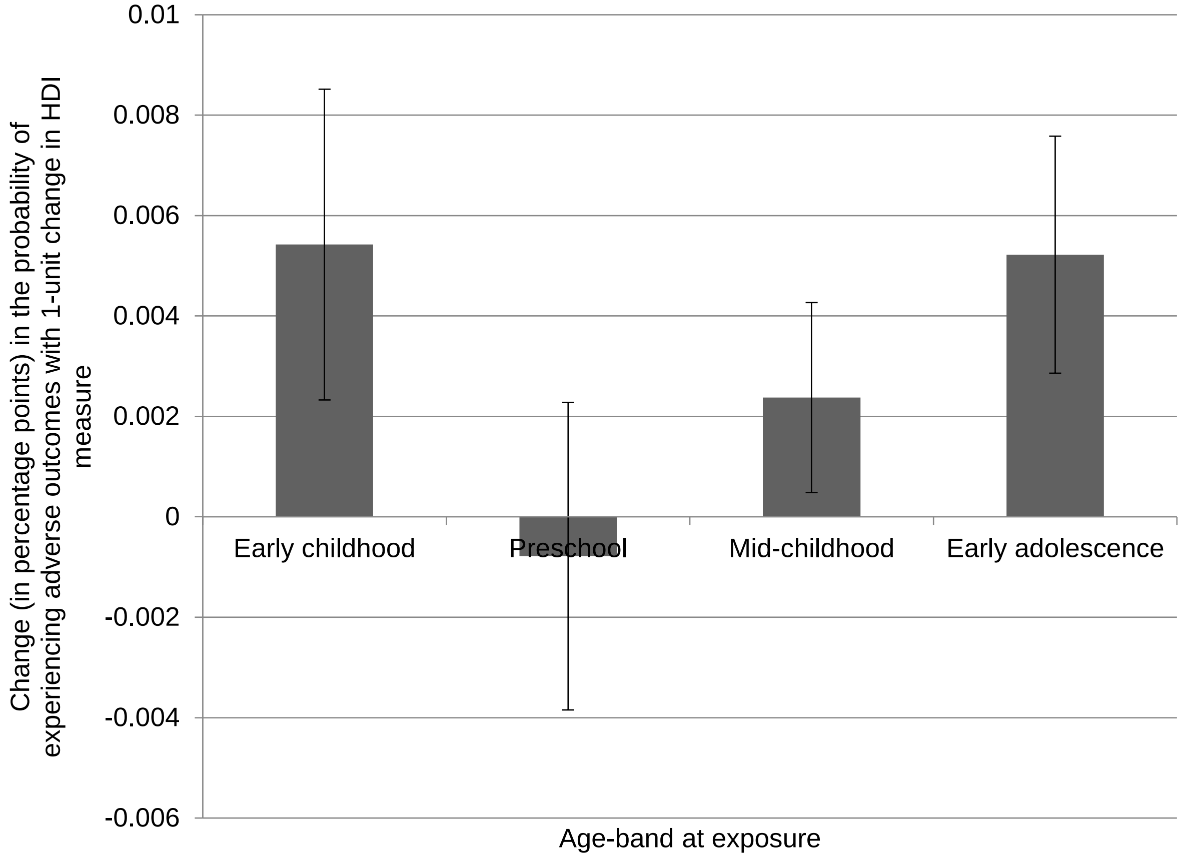

The model includes the indicators of HDI exposure in the four age-bands, birth year, birth order and gender. Error bars indicate 95% CIs

© 2021 Andersen SH. *JAMA Network Open.*

eFigure 7: Age-specific associations, by subgroups defined by mother's age at childbirth (N=605,344)

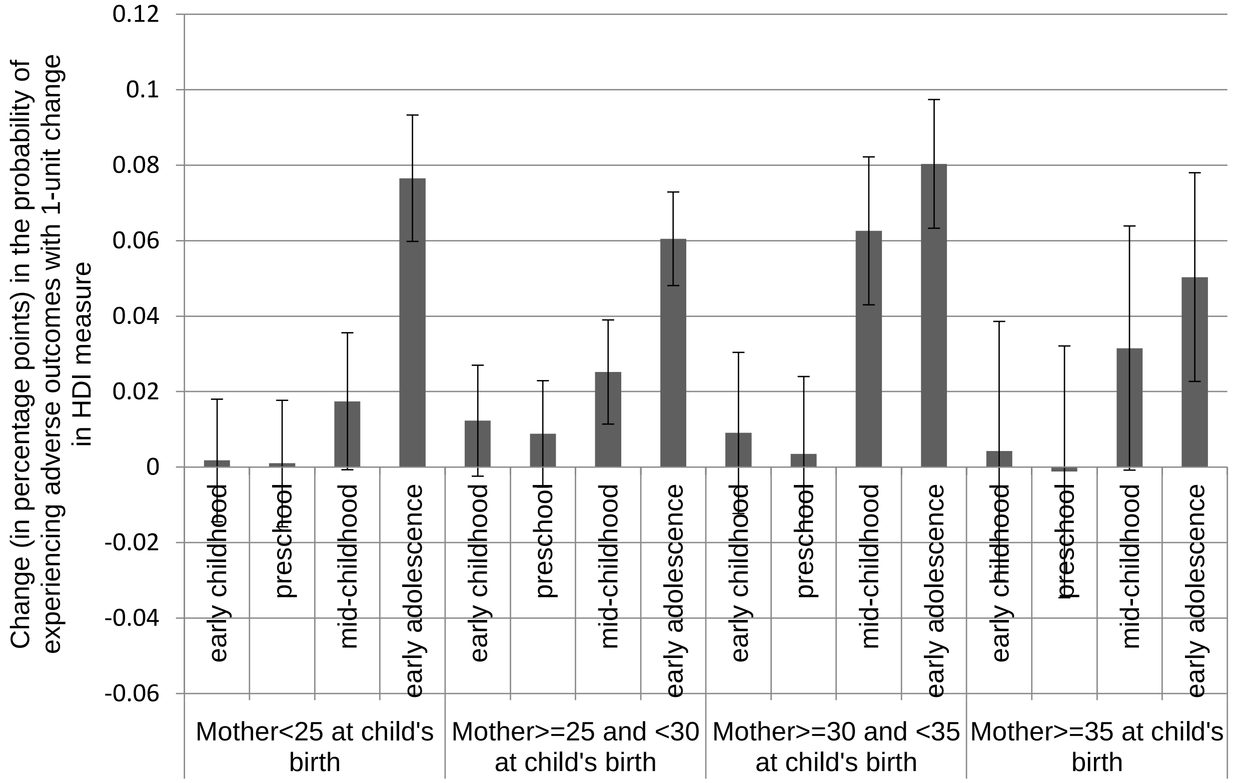

The model includes the indicators of HDI exposure in the four age-bands, birth year, birth order and gender. Error bars indicate 95% CIs

© 2021 Andersen SH. *JAMA Network Open.*
